# Scientific Items

**Published:** 1879-04-20

**Authors:** 


### SCIENTIFIC ITEMS.

**Pregnancy in the Elephant.**—We learn from N. Y. Medical Record that at the invitation of Mr. Tule, the manager of the "Great London Circus," a number of medical gentlemen visited the winter quarters of the circus at Twenty-third Street and Columbia Avenue, in Philadelphia, on Friday, March 14th, in order to inspect a female elephant said to be pregnant.

The female elephant believed to be pregnant had been covered twice by a male elephant at Concord, New Hampshire, on the 25th of May, 1878. At the time of examination she was consequently in the tenth month of pregnancy.

The two breasts, which are situated immediately between the front legs in the elephant, were full and swollen. The nipples were very prominent, and pointed outwards and downwards. The milk veins on the surface of the abdomen were also very prominent.

The general opinion hitherto held with regard to the act of copulation on the part of the elephant has been that it is performed while the female lies on her back.

In this instance, however, the act of copulation had been twice performed, and on both occasions the male had mounted upon the back of the female, and the penis, instead of curving backwards as is the case under artificial sexual excitement, had curved forwards and upwards. These facts were very thoroughly authenticated. This proves that previous ideas upon this subject have been altogether erroneous.

Curious glands, sebaceous in origin, were pointed out by the keeper in the roof of the elephant's mouth and behind the eyes. In the glands behind the eyes, wax is wont to accumulate, and causes much annoyance to the elephant unless removed.

It was further shown that the tongue of the elephant does not possesses any frænum.

As the period of normal pregnancy in the elephant is twenty months, it was expected that the calf would be born, if nothing unforeseen should occur, on January 25th, 1880. The birth of an elephant calf away from India is a great rarity.

**Experiment to Illustrate Geological Processes.**—M. de Chancourtois, professor of geology at the School of Mines in Paris, has devised an experiment to illustrate the formation of ridges of hills by risings in the crust of the globe while still soft from the primitive heat. M. de Chancourtois represents the globe by an inflated rubber ball, which takes the place of the terrestrial nucleus in fusion, destined to contract on cooling, and, by its shrinking, to produce on the surface the elevations and depressions which constitute the mountains and valleys of the existing earth. The ball is dipped in a bath of melted wax, and, when that has partially set, a small portion of the air is allowed to escape by the tap. The surface of the sphere then contracts slightly, and sinks in portions, wrinkling the wax. When a little more air is allowed to escape, these slight wrinkles become more distinct, and form ridges and hollows precisely analogous to the ranges of hills and valleys on the face of our planet, except that their relief is relatively greater. M. de Chancourtois lately presented to the Academy of Sciences two of these balls,—one in the first stage of the operation, and the other in the second or final one.—*Journal of Chemistry.*

**A Hairy Water Tortoise.**—Mr. Frank Buckland, in Land and Water, describes a curious water tortoise from China that had been presented to him. It is a terrapin which apparently has hairs growing out from its back. These apparent hairs consist simply of some water grass, something like the weedy material growing in decaying wood-work and lock gates of rivers. Whether the growth is produced artificially by the Chinese, or whether it is natural, is not known.—*Sci. Am.*

**Caloptine.**—A chemical examination of the dead Rocky Mountain locusts, by the United States Entomological Commission, shows that these insects furnish a new oil, which will be christened caloptine, and a very large percentage of pure formic acid. Though this acid exists in the ant and some other insects, it is with difficulty obtained in large quantities; whereas by the action of sulphuric acid upon the locust juices, it passes off with great readiness, and in remarkable quantity and gravity.—*Ec. Med. Journal.*

**Absorption of Carbonic Oxide by Living Organisms.**—N. Grehaut has experimented with mixtures of air and minute portions of cabonic oxide. He finds that a man or an animal, when compelled for half an hour to breathe an atmosphere containing only 1-779 of carbonic oxide, absorbs that gas in sufficient quantities to saturate about half of the red globules of the blood, so that they become incapable of absorbing oxygen. In an atmosphere containing 1-1449 of carbonic oxide, about a quarter of the red globules are similarly saturated. These results are interesting and important in relation to physiology and hygiene.—*Journal of Chemistry.*

**Meteorology for 1878.**—It is said that the year 1878 was not only the warmest of the last ten, but also that by far the greatest amount of rain fell. With such an excess of rain (11 inches above the average) one should expect a much cooler year than usual. Other causes must have counteracted this influence.

It will also be noticed that the whole yearly range of temperature, during the last fifteen years, has been only 7.72°, while the amount of rain in the last ten years has varied more than twenty-three and a half inches (23.59).

**Self-Luminous Clocks.**—The new Swiss self-luminous clock dials are rendered visible in the dark by covering the figures with the sulphide of calcium, which has long been noted for its phosphorescence. Its remarkable qualities in this respect are evident from the fact that it was visible in total darkness after having been closely shut up in a box for five days.

**Disappearance of Nebulæ.**—Two nebulæ of the Southern heavens which were described by Herchel, and which have long been prominent celestial objects observed by astronomers, have, of late, entirely disappeared from view, even through the Melbourne reflector.

**Invisible Ink.**—Bottger recommends as an invisible ink for postal cards sulphuric acid diluted with fifty parts of water, a quill pen being used for the writing, which is invisible until heated, when it becomes black.—*Journal of Chemistry.*

